# DNA damage response-related ncRNAs as regulators of therapy resistance in cancer

**DOI:** 10.3389/fphar.2024.1390300

**Published:** 2024-08-26

**Authors:** Ziru Gao, Xinchi Luan, Xuezhe Wang, Tianyue Han, Xiaoyuan Li, Zeyang Li, Peifeng Li, Zhixia Zhou

**Affiliations:** Institute for Translational Medicine, The Affiliated Hospital of Qingdao University, Qingdao Medical College, Qingdao University, Qingdao, China

**Keywords:** DNA damage response (DDR), non coding RNA (ncRNA), resistance, radiotherapy, chemotherapy

## Abstract

The DNA damage repair (DDR) pathway is a complex signaling cascade that can sense DNA damage and trigger cellular responses to DNA damage to maintain genome stability and integrity. A typical hallmark of cancer is genomic instability or nonintegrity, which is closely related to the accumulation of DNA damage within cancer cells. The treatment principles of radiotherapy and chemotherapy for cancer are based on their cytotoxic effects on DNA damage, which are accompanied by severe and unnecessary side effects on normal tissues, including dysregulation of the DDR and induced therapeutic tolerance. As a driving factor for oncogenes or tumor suppressor genes, noncoding RNA (ncRNA) have been shown to play an important role in cancer cell resistance to radiotherapy and chemotherapy. Recently, it has been found that ncRNA can regulate tumor treatment tolerance by altering the DDR induced by radiotherapy or chemotherapy in cancer cells, indicating that ncRNA are potential regulatory factors targeting the DDR to reverse tumor treatment tolerance. This review provides an overview of the basic information and functions of the DDR and ncRNAs in the tolerance or sensitivity of tumors to chemotherapy and radiation therapy. We focused on the impact of ncRNA (mainly microRNA [miRNA], long noncoding RNA [lncRNA], and circular RNA [circRNA]) on cancer treatment by regulating the DDR and the underlying molecular mechanisms of their effects. These findings provide a theoretical basis and new insights for tumor-targeted therapy and the development of novel drugs targeting the DDR or ncRNAs.

## 1 Introduction

Cells constantly respond to DNA damage caused by both endogenous and exogenous stimuli, such as replication pressure, telomere shortening, reactive oxygen species (ROS), ultraviolet radiation, ionizing radiation, and chemical toxins (Goldstein and Kastan, 2015). This process is known as DNA damage repair or the DNA damage response (DDR) and is defined as the cell’s response to DNA damage. DDR is a complex repair mechanism network composed of signaling pathways that maintain the stability and integrity of mammalian genomes (Li et al., 2024; Zuo et al., 2024). Based on the different types of DNA damage, DDR initiates repair processes through different pathways, such as mismatch repair (MMR), base excision repair (BER), nucleotide excision repair (NER), homologous recombination repair (HRR), and nonhomologous terminal junction (NHEJ) pathways (Liu et al., 2024) (Thote et al., 2024)^.^ Activation of the DDR often requires close coordination of these different DNA repair pathways, as well as signal transduction associated with cell cycle arrest, to allow for DNA damage repair and to prevent DNA damage from being replicated or transmitted to the next-generation (Ashwell and Zabludoff, 2008; Jackson and Bartek, 2009). Dysregulation of the DNA damage response (DDR) has been proven to be associated with susceptibility to the development of various diseases, including cancer, and can also lead to hypersensitivity or tolerance to treatment, which can be used to improve clinical treatment (Jurkovicova et al., 2022).

Choosing appropriate therapies and medication regimens remains a major challenge in cancer treatment. Chemotherapy and radiotherapy, which rely on cytotoxic DNA damage effects, are still the first-line treatment methods for many unresectable or metastatic malignant tumors. However, due to the unique strong adaptability of cancer cells, the DDR can be dysregulated, leading to hypersensitivity or drug resistance reactions against cancer agents in cancer cells (Jurkovicova et al., 2022). Resistance is believed to arise through genetic alterations and non genetic mechanisms, without altering the DNA sequence to reduce sensitivity to different cancer cells (Brauner et al., 2016; Rao and Ayres, 2017). A defective DDR drives cancer progression and recurrence by preselecting subclones with intrinsic or acquired drug resistance, leading to the development of tumor heterogeneity (Jurkovicova et al., 2022; Lytle et al., 2018; Bakhoum and Landau, 2017). At least 330 genes are involved in the imbalance of the DDR in tumor cells and are valuable targets for new cancer treatment methods (Vogelstein et al., 2013). These DDR-related genes have laid the foundation for a large number of new DDR inhibitors (DDRis) and DDR-based strategies. Recently, several DDRis, such as inhibitors of poly (ADP-ribose) polymerase (PARP), ataxia telangiectasia mutated (ATM) and ataxia–telangiectasia-mutated and Rad3-related kinase (ATR), have been identified as potential alternative strategies for overcoming cancer resistance, and their combination with radiation, cytotoxicity, or targeted drugs can maximize the benefits of DDR-targeted therapy (Cheng et al., 2022; Qiu et al., 2022). More importantly, recent studies have shown that DDR-related genes include not only protein-coding genes, such as kinases and pathway regulatory proteins but also noncoding genes, such as noncoding RNAs (ncRNAs).

ncRNAs have become important regulatory factors for gene expression by influencing chromatin structure, DNA/RNA/protein modifications, molecular stability, and RNA splicing (Tian et al., 2019).They are not only involved in physiological processes such as cell development and differentiation but also participate in the pathological processes of various diseases, especially tumors (Esteller, 2011; Rinn and Chang, 2012; Liu et al., 2023; Ao et al., 2023). Increasing evidence suggests that ncRNAs, especially microRNAs (miRNAs), long noncoding RNAs (lncRNAs), and circular RNAs (circRNAs), are abnormally expressed in various cancers and are associated with the occurrence, development, and metastasis of tumors, as well as the acquisition of tumor resistance to treatment (Liu et al., 2022a; Zhou et al., 2022). They play important roles in tumor treatment resistance and tolerance by controlling multiple signaling pathways in cells, including the cell cycle, proliferation, apoptosis, DDR, and other key cellular signaling pathways, as well as regulating the expression of therapeutic targets or genes involved in therapeutic metabolism or transport (Fanale et al., 2016; Wei et al., 2020; Matsui and Corey, 2017; Xu et al., 2021). Moreover, the inhibition or overexpression of these ncRNAs related to tumor treatment has been demonstrated to regulate the activation or inactivation of their targeted oncogenes, tumor suppressor genes, or certain regulatory genes, possibly reversing the treatment tolerance of tumor cells and improving their sensitivity to therapeutic agents (Chen B. et al., 2022; Zhang et al., 2020; Liu et al., 2022b). Therefore, identifying and understanding ncRNAs and their mechanisms of action related to tumor treatment response can not only identify potential candidate molecules for predicting treatment response, but also develop personalized and more targeted treatment plans for cancer patients (Kutashev et al., 2024; Mamontova et al., 2024).

In this review, we first provide an overview of the basic information on the DDR (including major checkpoint kinases and repair pathways), as well as the regulatory roles of ncRNAs in the tolerance or sensitivity to tumor chemotherapy and radiation therapy. We focused on the impact of ncRNAs (mainly miRNAs, lncRNAs, and circRNAs) on cancer treatment through the regulation of the DDR. These findings provide both a theoretical basis for research on molecular markers or targeted therapies related to cancer treatment based on the DDR or ncRNAs and a scientific basis for rational planning of clinical medication and the development of innovative drugs.

## 2 The DDR in cancer therapy resistance

Tumor therapy resistance is mainly related to the intrinsic sensitivity of tumor cells and their microenvironment (Hu and Jiang, 2019). Therefore, different tumors exhibit different sensitivities or tolerances to different treatment methods, resulting in different treatment outcomes. At present, the factors related to therapeutic resistance mainly include cell hypoxia, the cell cycle, cell apoptosis, and the DDR (Bacová et al., 2000). In recent years, an increasing number of studies have focused on the role of the DDR in cancer therapy resistance. Many studies have shown that the DDR is a key inducer of drug resistance in cancer cell therapy, and alterations in the DDR of tumor cells commonly contribute to emerging resistance to tumor therapy (Eich et al., 2013; McMullen et al., 2020; Wang S. et al., 2022; Lim and Ma, 2019). Alterations in the DDR lead to genomic instability and the production of new antigens, upregulating the expression of programmed death ligand 1 (PD-L1), also known as differentiation cluster (CD) 279, which is an important immunosuppressive molecule (Liu et al., 2022b). PD-L1 interacts with cGAS-STING (cGAS-STING) signals, like interferon genes, to suppress immune responses and promote cancer cell tolerance by inhibiting T cell inflammation (Jiang et al., 2021). Moreover, many related genes or pathways are involved in the molecular mechanisms of tumor treatment tolerance mediated by the DDR and may vary depending on the treatment method.

### 2.1 The main checkpoint kinases involved in the DDR

**FIGURE 1:**
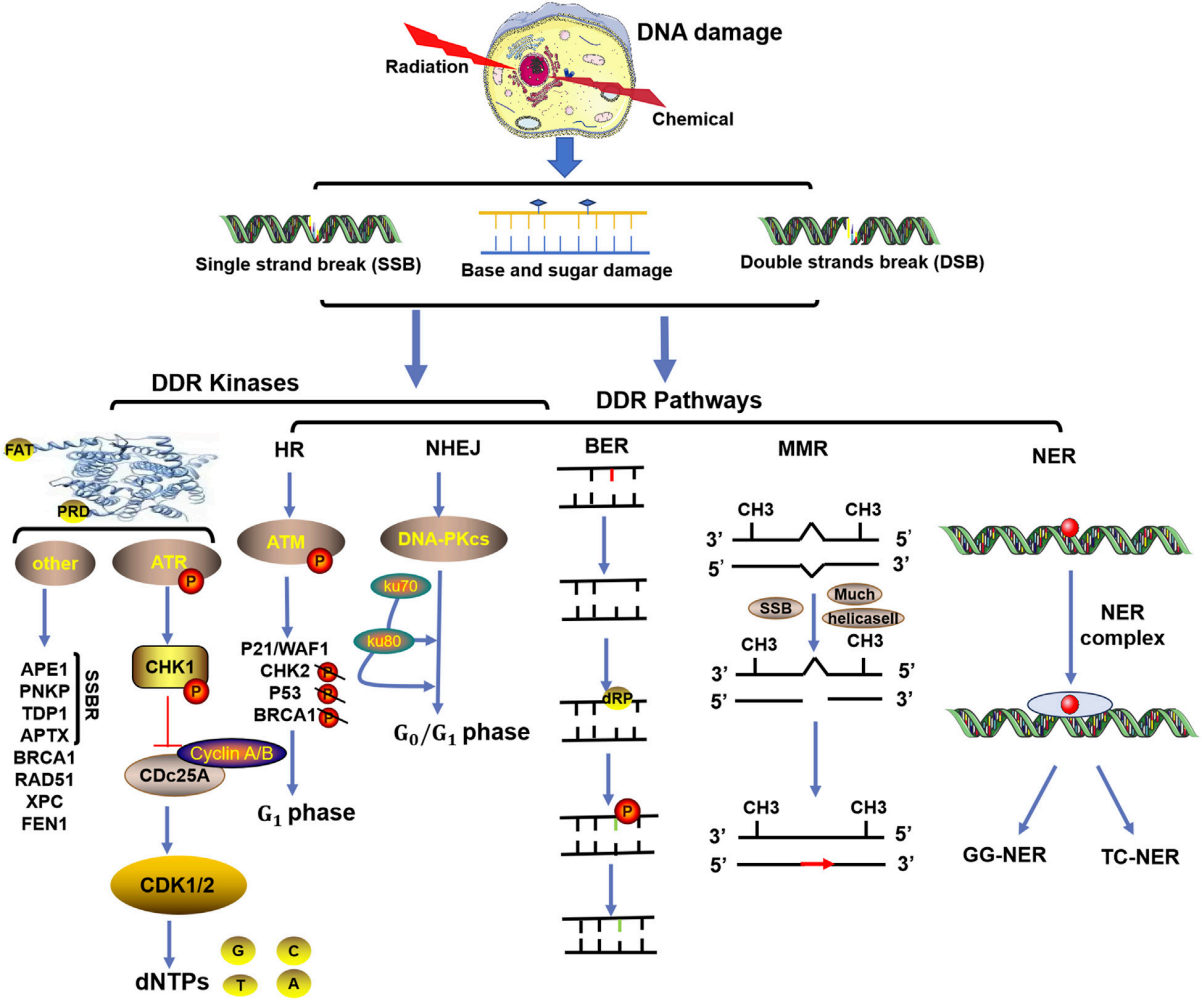
Functional complexes of DNA damage response (DDR) kinases and repair signaling pathways activated in response to radiation- or chemical-induced DNA damage. The three major types of DNA damage induced by radiation or chemical drugs include base and sugar damage, single-strand breaks (SSBs), and double-strand breaks (DSBs). In response to DNA damage, the rapid activation of checkpoint kinases (mainly ATM, ATR and DNA-PK) is the first signal transduction wave to be transmitted by cells. Based on the different types of DNA damage, the DNA damage response (DDR) initiates repair processes through different pathways, such as homologous recombination repair (HR), nonhomologous terminal junction (NHEJ), base excision repair (BER), mismatch repair (MMR), and nucleotide excision repair (NER).

In response to DNA damage, the rapid activation of checkpoint kinases is the first signal transduction wave to be transmitted by cells, as shown in Figure 1. In vertebrate cells, at least three major protein kinases, namely, ATM, ATR and DNA-dependent protein kinase (DNA-PK), are associated with DDR (Blackford and Jackson, 2017). These kinases share similar upstream frap–atm–trap (FAT) domains and downstream phosphatidylinositol 3-kinase-like protein kinase (PIKK) regulatory domains (PRD). These proteins usually phosphorylate a serine or threonine residue followed by glutamine but can also be autophosphorylated (Blackford and Jackson, 2017). Moreover, to prevent the harmful effects of DNA single-strand break repair (SSBR) damage, which is one of the most common DNA lesions, cells are equipped with specialized enzymes, including apurinic/apyrimidinic (AP) endonuclease 1 (APE1), polynucleotide kinase-phosphatase (PNKP), tyrosine DNA phosphodiesterase 1 (TDP1), apatasin (APTX), and DNA ligases (Iyama and Wilson, 2013). These enzymes can produce appropriate 3′and 5′termini to cause chain breaks and interruptions (Iyama and Wilson, 2013). In addition, several other kinases related to cell vulnerability, such as breast-cancer susceptibility gene 1 (BRCA1), radiation sensitive 51 (RAD51), RAD52, xeroderma pigmentosum group C (XPC), and flap endonuclease 1 (FEN1), are involved in the DDR (Li E. et al., 2022). Among these modifications, ATM plays an important role in the response to DNA double-strand break (DSB) damage induced by exogenous sources such as radiation or anticancer chemotherapeutic agents (Shiloh, 2001). ATM is a product of a gene mutated in human hereditary ataxia degenerative disorder that belongs to the phosphatidylinositol 3-kinase superfamily. The activation of ATM can lead to the induction of p21/WAF1, the inhibition of cyclin-dependent kinase activity, and the failure to phosphorylate key substrates such as checkpoint kinase 2 (CHK2), p53, and BRCA1, ultimately leading to G1 arrest (Lavin, 1999; Jin and Oh, 2019). Correspondingly, abnormal ATM activation is often one of the key factors through which tumor cells develop tolerance to treatment (Jin and Oh, 2019; Choi et al., 2016). However, the inhibition of ATM increases the sensitivity of cancer cells to radiotherapy or chemotherapy (Lavin and Yeo, 2020). Therefore, several ATM inhibitors, such as KU-559933, CP-466722, AZ32, and AZD1390, have been developed and evaluated as anticancer agents in clinical applications (Jin and Oh, 2019).

In contrast to ATM, which is activated by DSBs, ATR proteins respond to SSBs. ATR is essential for cell proliferation, and one of its important functions is to activate checkpoint kinase 1 (CHK1). CHK1 degrades the phosphatase cell division cycle 25 A (Cdc25A) and inhibits cyclin-dependent kinases (CDKs), thereby arresting cell cycle progression and providing time for DNA repair (Blackford and Jackson, 2017). When challenged by chemotherapeutic drugs and/or radiation, the cell cycle checkpoint protein ATR and its major downstream effector CHK1 prevent the entry of cells with damaged or imperfectly replicated DNA into mitosis (Jin and Oh, 2019). This regulation is particularly noticeable in cells with a compromised G1 checkpoint, a characteristic frequently present in cancer cells (Jin and Oh, 2019). Additionally, ATR and/or CHK1, especially in cells with active oncogenes, limit excessive origin firing to minimize replication stress (RS), making them excellent therapeutic targets (Jin and Oh, 2019; Qiu et al., 2018; Lecona and Fernandez-Capetillo, 2018). The inhibition of ATR/CHK1 can be achieved by blocking cell cycle checkpoints, enhancing the killing effect of cytotoxic drugs or radiotherapy on tumor cells (Ma et al., 2011). At present, ATR/CHK1 inhibitors, such as UCN-01 (7-hydroxystaurosporine), AZD7762, LY2603618, and PF-00477736, have been developed for p53-deficient cells and have been used as single drugs or in combination with radiotherapy or multiple genotoxic chemotherapies in preclinical and clinical studies (Qiu et al., 2018).

Along with ATM and ATR, DNA-PK plays a key role in the DDR and is a multiprotein complex consisting of a catalytic subunit (DNA/PK) and the regulatory heterodimer Ku (Ku70/Ku80) (Yue et al., 2020). Like ATM, DNA-PKcs is activated by DSBs but stabilizes and aligns DNA ends to play an important role in maintaining genomic integrity (Damia, 2020). The interaction between two DNA-PKs located at the end of each DSB activates their intrinsic protein kinase activity, resulting in self-phosphorylation and dissociation of DNA-PK to activate the endonuclease and recruit different processing factors involved in the G0/G1 phases of the cell cycle (Medová et al., 2020). Abnormal expression of DNA-PK was shown to be beneficial for tumor development, progression, and treatment tolerance-related metastasis by regulating cell survival signaling pathways (Goodwin and Knudsen, 2014; George et al., 2019). Mutations and deletions in DNA PKcs are associated with enhanced chromosomal exchange and increased sensitivity to reagents that cause DNA DSBs (van der Burg et al., 2009; Woodbine et al., 2013). Therefore, many types of DNA-PK inhibitors, such as small-molecule substances, nucleotides, antisense oligonucleotides, and small interfering RNAs, have been developed and combined with chemical drugs to enhance the drug sensitivity of tumors (Damia, 2020). For example, the DNA-PK inhibitor AZD7648, developed by Jacqueline H.L. Fok, has been shown to be powerful and selective and to increase the effectiveness of both doxorubicin and irradiation. Additionally, the DNA-PK inhibitor olaparib has been approved for a number of applications in the treatment of breast and ovarian cancers (Fok et al., 2019).

### 2.2 The main repair pathways involved in the DDR

Generally, DNA repair systems are divided into direct and indirect repair systems. The direct repair system is involved in the repair of DNA during replication and is mediated by direct proteins catalyzed by the main DNA polymerase. O6 methylguanine-DNA methyltransferase (MGMT) repairs endogenous and alkylating agent-induced damage to the guanine O6 site and ultimately repairs pyrimidine dimers (Iyama and Wilson, 2013). The indirect DNA repair system includes at least five pathways, such as the MMR, NER, BER, HR, and NHEJ pathways (Iyama and Wilson, 2013), as shown in Figure 1. Among them, the HR and NHEJ pathways are involved in the response of double-strand break repair (DSBR) to SBRs. The HR pathway includes ATM as the core component and plays roles in cell and S phase division because it requires homologous sister chromatids to execute. NHEJ, with DNA-PK as its core component, can play roles in both dividing and nondividing cells and is independent of the cell cycle (Iyama and Wilson, 2013). Defects in HR or NHEJ can lead to the occurrence and development of many human diseases, including sensitivity to radiation and drug therapy for cancer (Difilippantonio et al., 2002).

BER is the most important repair pathway in the DNA repair system and has been proven to be related to the incidence of various malignant tumors. In the BER pathway, damaged bases are recognized and removed by DNA glycosidases, after which DNA damage is repaired by polymerase and nuclease (Grundy and Parsons, 2020). A significant portion of the DNA damage caused by external (photons and particle beams) and internal radiation technologies is the repair target of the BER pathway (Grundy and Parsons, 2020). For cancer treatment to be effective, the amount of DNA damage caused by radiotherapy must be greater than the repair ability of cancer cells. Recent studies have shown that PARP inhibitors (PARPis) promote the effectiveness of combination therapy with anti-PD-1 combined with ionizing radiation in colorectal cancer models, while PD-L1 expression is inversely proportional to BER gene expression, including the core genes eight-oxoguanine-DNA glycosylase (OGG1) and APE1 of the BER pathway (Permata et al., 2019).

The MMR pathway plays a crucial role in maintaining DNA replication fidelity and genomic stability; this pathway mainly involves detecting mismatches and inserting or deleting rings, inducing single-stranded incisions, and subsequently facilitating DNA repair through polymerase, nuclease, and ligase (Jiang et al., 2021). The two core protein complexes of the MMR pathway are MutSα (heterodimer of the MutS homolog 2 [MSH2] and MutS homolog 6 [MSH6] proteins) and MutS β (heterodimer of the MSH2 and MSH3 proteins), which can bind to mismatches in an adenosine triphosphate-dependent manner and subsequently recruit MutL α (heterodimer of the mutL homolog 1 [MLH1] and postmeiotic segregation increased 2 [PMS2] proteins) (Liu et al., 2017). Research has confirmed that MMR deficiency is a molecular characteristic that leads to microsatellite instability (MSI) and induces cancer (Koessler et al., 2008). Temozolomide (TMZ) and radiation therapy have antitumor effects on glioblastoma (GBM) mainly through ineffective MMR pathway activity and apoptosis induction (Ao et al., 2023). Research has shown that the MGMT pathway can repair DNA damage caused by TMZ, but GBM patients treated with TMZ may benefit from tumor cells that do not express the MGMT protein. Patients with low or missing MGMT expression levels still exhibit drug resistance. After MGMT deletion, O6 guanine remains in DNA and undergoes mismatch base pairing with thymine during DNA replication, ultimately leading to a lack of MMR (Li et al., 2023).

The NER pathway can handle spiral distortion lesions through two NER subpathways, which typically cleave 22 to 30 bases of oligonucleotides to eliminate damage, triggering the production of single-stranded DNA (ssDNA) (Li et al., 2023). One of the subpathways is the global genome NER (GG-NER), which is initiated by the HR23B/XPC complex, and distorted lesions are identified throughout the entire genome. Another pathway is transcriptional coupled repair (TCR), which plays a role in preventing damage to extended RNA polymerase (Hoeijmakers, 2001; Curtin, 2013). Genetic defects in the NER gene are the foundation of three serious genetic diseases, xeroderma pigmentosum (XP), Cockayne syndrome (CS), and lignosulfia malnutrition (TTD), which are associated with a high risk of skin cancer (Butkiewicz et al., 2002). Research has shown that the upregulation of NER genes may be an important reason for drug (such as cisplatin) resistance in some clinical cancer chemotherapeutic agents, such as ovarian and bladder cancers (Saldivar et al., 2007; Mouw, 2017).

Besides all these pathways and specific checkpoint kinase, nrecent studies have shown that ncRNAs can also participate in DDR by regulating the expression of oncogenes or suppressor genes during tumorigenesis. Moreover, different types of ncRNAs can target specific factors or pathways of different DDR, promoting or inhibiting tumor drug resistance, as described below.

## 3 Noncoding RNA regulation in cancer therapy resistance

**FIGURE 2:**
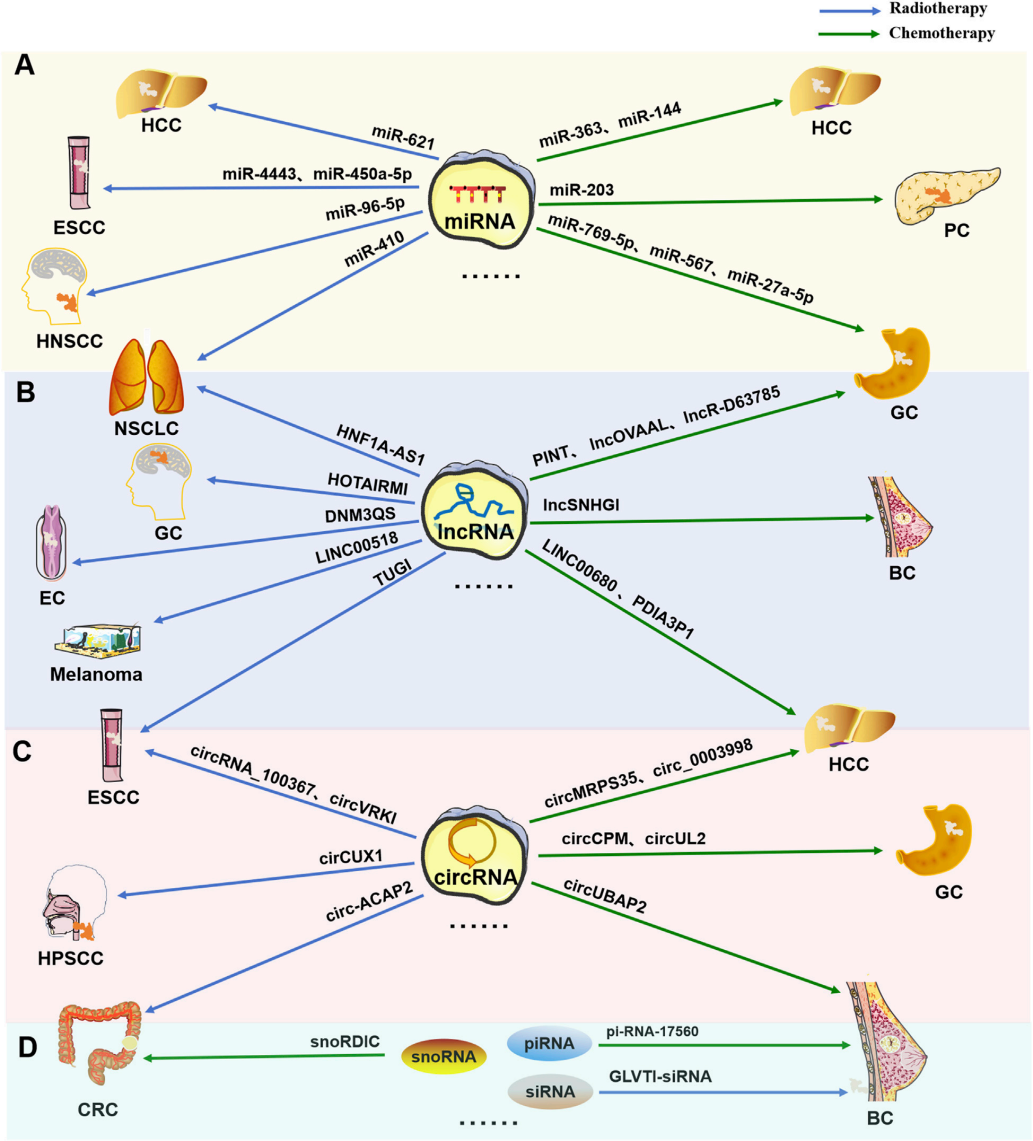
Multiple types of ncRNAs are related to tumor radiotherapy or chemotherapy tolerance. Typical tumor radiotherapy (blue arrow) or chemotherapy (green arrow) tolerance-related miRNAs **(A)**, lncRNAs **(B)**, circRNAs **(C)**, and other types of RNAs (including snoRNAs, piRNAs, and siRNAs) **(D)**.

ncRNAs, especially miRNAs, lncRNAs and circRNAs, have long been widely recognized as universal regulatory factors for various cancer characteristics, such as proliferation, apoptosis, invasion, and metastasis. However, for tumors, resistance or tolerance to chemotherapy, radiation therapy, targeted therapy, and immunotherapy remains a major setback. Recent studies have shown that ncRNAs play important roles as drivers of oncogenes or tumor suppressor genes to regulate cell proliferation or death signals in patients resistant to different cancer therapies, such as radiotherapy and chemotherapy, as shown in Figure 2.

### 3.1 Regulation of miRNAs in tumor therapy resistance

miRNA is a type of short noncoding RNA with a length of approximately 19–25 nucleotides that can interact with the 3′ untranslated region (3′-UTR) of its target gene to regulate post transcriptional mechanisms of gene expression (Rykova et al., 2022). In recent years, an increasing number of miRNAs have been shown to be closely related to the resistance of tumors to radiotherapy and chemotherapy, as shown in Supplementary Table S1 and Supplementary Table S2.

#### 3.1.1 Regulation of cancer chemotherapy resistance

There are numerous drugs that can treat multiple cancers, such as cisplatin (CDDP), 5-fluorouracil (5-FU), and doxorubicin (DOX). Correspondingly, many tumors have developed resistance to these spectral anticancer drugs. Moreover, some small RNAs have been reported to be associated with tolerance to these drugs. For example, researchers have demonstrated a significant decrease in miR-363, which leads to the release of its target gene myeloid cell leukemia 1 (Mcl-1) and enhances tumor resistance to CDDP (Ou et al., 2015). MiR-769-5p is upregulated in drug-resistant cancer (GC) cells and can target caspase-9 (CASP9) inactivation through exosomes, mediate the direct degradation of the p53 protein via the E3 ubiquitin ligase 4-like protein (NEDD4L), and ultimately induce resistance in CDDP-sensitive receptor cells (Jing et al., 2022). High expression of miR-203 targets DJ-1 mRNA and inhibit its expression, thereby negatively regulating the expression of chromosome ten (PTEN) and affecting the activity of the PTEN-phosphatidylinositol-3 kinase (PI3K)/protein kinase (AKT) pathway to influence proliferation, apoptosis, and drug resistance in pancreatic cancer cells (Du et al., 2019). In 5-FU-resistant tumors, miR-144 is significantly downregulated, and its upregulation may reverse the acquisition of 5-FU resistance by inhibiting the nuclear factor-erythroid 2-related factor 2 (Nrf2)-dependent pathway (Zhou et al., 2016). miR-567 is also significantly downregulated in cancer cells, and its upregulation can reduce drug resistance through the phosphoinositol-3-kinase adaptor protein 1 (PIK3AP1)–PI3K/AKT–c-MYC (MYC) feedback loop (Zhang F. et al., 2019). For Dox-resistant tumors, the overexpression of miR-27a-5p reportedly enhances the drug sensitivity of tumor cells through sarcoma (RAS)/mitogen-activated protein kinase (MEK)/c-FOS (FOS) and PTEN/AKT/SMAD family member 1 (SMAD1) pathway dependence (He et al., 2021).

#### 3.1.2 Regulation in cancer radiotherapy resistance

Some miRNAs have been shown to be associated with tumor radiation resistance in various cancers and can affect treatment efficacy through different molecular pathways. miR-96-5p was found to promote the resistance of head and neck squamous cell carcinoma (HNSCC) to radiation therapy by inhibiting the expression of PTEN (Vahabi et al., 2019). miR-4443 is significantly upregulated in radiation-resistant esophageal squamous cell carcinoma (ESCC) cells and can promote cell radioresistance by targeting the protein tyrosine phosphatase receptor type PTPRJ (Shi et al., 2022). In hepatocellular carcinoma (HCC), the downregulation of miR-621 leads to an upsurge in SET Domain, Bifurcated 1 (SETDB1) activity, thereby augmenting gene silencing that impacts growth and DNA damage responses (Shao et al., 2019). Conversely, overexpressing miR-621 targets SETDB1, diminishing its functionality and resulting in increased expression of genes pivotal for DNA repair, apoptosis, and cell cycle regulation (Shao et al., 2019). Yue Yuan et al. reported that overexpression of miR-410 can enhance radioresistance in non-small cell lung cancer (NSCLC) by targeting the PTEN/PI3K/mTOR axis, suggesting that miR-410 is a therapeutic target for cancer (NSCLC) radiotherapy (Yuan et al., 2020). It was reported that overexpression of miR-450a-5p enhanced radiosensitivity by targeting bispecific phosphatase 10 (DUSP10) in ESCC (Dai et al., 2020). DUSP10 is associated with negative regulation of the mitogen activated protein kinase (MAPK) signaling pathway which plays a crucial role in cellular processes such as proliferation, differentiation, and response to DNA damage (Dai et al., 2020). That is to say, miR-450a-5p may enhance the activation of the MAPK pathway by downregulating DUSP10, thereby increasing sensitivity to radiotherapy by promoting apoptosis and inhibiting cell proliferation to cope with DNA damage, suggesting that miR-450a-5p is a potential radiosensitizer.

### 3.2 Regulation of lncRNAs in tumor therapy resistance

Unlike miRNAs, lncRNAs are a class of ncRNAs with lengths exceeding 200 bp that interact with DNA, RNA, and proteins to regulate gene expression (Xing et al., 2021; Tan et al., 2021). They also play important roles in tumor chemotherapy and radiation tolerance (McCabe and Rasmussen, 2021), as shown in Supplementary Table S1 and Supplementary Table S2.

#### 3.2.1 Regulation in cancer chemotherapy resistance

Like miRNAs, lncRNAs have also been reported to be involved in the resistance of various tumors to spectral antitumor drugs, such as the cisplatin lncRNA SNHG1, which is significantly upregulated in breast cancer (BC) cells, and its silencing enhances the sensitivity of tumor cells to CDDP (Zhang M. et al., 2021). The lncRNA LINC-PINT is downregulated in CDDP-resistant GC cells, and its overexpression can enhance cell drug sensitivity by regulating the enhancer of zeste homolog 2 (EZH2)/autophagy-related gene 5 (ATG5) axis (Zhang C. et al., 2022). LINC00680 can activate AKT3 by absorbing miR-568 to reduce tumor cell resistance to 5-FU, which may lead to potential therapeutic targets (Shu et al., 2021). The lncRNA OVAAL could promote 5-FU resistance in GC cells by promoting pyrimidine biosynthesis (Tan et al., 2022). The lncRNA PDIA3P1, which is a component of the homolog of mRNA transport mutant 4 (hMTR4)-protein disulfide isomerase family A member three pseudogene 1 (PDIA3P1)/miR125/124–tumor necrosis factor receptor-associated factor 6 (TRAF6) axis, was upregulated in HCC cells treated with Dox (Xie et al., 2020). In addition, our previous research revealed that the lncRNA D63785 was highly expressed in GC tissues and cells and that its inhibition could increase the sensitivity of GC cells to Dox by upregulating the expression of miR-422a and inhibiting myocyte enhancer factor 2D (MEF2D) (Zhou et al., 2018).

#### 3.2.2 Regulation in cancer radiotherapy resistance

Ulvi Ahmadov et al. reported that the lncRNA HOTAIRM1 was highly expressed in glioblastoma cells. HOTAIRM1 can reduce radiosensitivity by regulating mitochondrial function and ROS levels in glioblastoma cells (Ahmadov et al., 2021). Hongfang Zhang et al. reported that the lncRNA DNM3OS was upregulated in esophageal cancer cells and could confer radioresistance by regulating the DNA damage response (Zhang H. et al., 2019). In ESCC cells, lncTUG1 expression was upregulated and enhanced cell radiation tolerance by reducing miR-144-3p levels and regulating the mesenchymal–epithelial transition factor (MET)/epidermal growth factor receptor (EGFR)/AKT axis (Wang et al., 2020). Wang Zhiyu et al. indicated that the lncRNA HNF1A–AS1 was upregulated in NSCLC cells. The overexpression of HNF1A–AS1 could significantly enhance radioresistance by regulating the expression of miR-92a-3p (Wang et al., 2021a). In addition, LINC00518 was reported to be significantly upregulated in melanoma and to target the miR-33a-3p/hypoxia-inducible factor 1 (HIF-1α) negative feedback loop to enhance the radioresistance of cells (Liu Y. et al., 2021).

### 3.3 Regulation of circRNAs in tumor therapy resistance

CircRNAs are also a large class of ncRNAs approximately 100 nt in length. These RNAs have a covalent closed loop structure but do not have a 5′cap or 3′poly-A tail (Kristensen et al., 2022; Kristensen et al., 2019). They also play important roles in gene expression at the transcriptional and posttranscriptional levels by mainly acting as miRNA sponges and protein scaffolds (Zhou et al., 2021). CircRNAs are the third largest class of endogenous ncRNAs and, like miRNAs and lncRNAs, are associated with tumor treatment tolerance.

#### 3.3.1 Regulation in cancer chemotherapy resistance

CircMRPS35 was highly expressed in HCC cells, and its encoded peptide (circMRPS35-168aa) promoted the resistance of tumor cells to CDDP (Li P. et al., 2022). CircUBAP2 was also significantly upregulated in triple-negative breast cancer (TNBC) cells, but it could reduce the resistance of tumor cells to CDDP by regulating the miR-300/anti-silencing function 1B (ASF1B) axis (Wang L. et al., 2022). In GC cells and tissues resistant to 5-FU, the expression of circCPM was significantly upregulated, which can play a role in 5-FU resistance by targeting the protein kinase AMP-activated α two catalytic subunit (PRKAA2) (Fang et al., 2022). For Dox resistance, circle-0003998 was highly expressed in HCC cells, but this resistance could be reduced by regulating the miR-218-5p/eukaryotic initiation factor-5A2 (EIF5A2) axis (Li X. et al., 2020). CircCUL2 was significantly downregulated in GC cells and tissues, and its overexpression could inhibit Dox resistance in tumor cells through miR-142-3p/Rho-associated coiled-coil-containing protein kinase (ROCK)-mediated autophagy activation (Peng et al., 2020).

#### 3.3.2 Regulation in cancer radiotherapy resistance

It has been reported that circRNA_100367 is downregulated in ESCC cells and can reduce radioresistance via the miR-217/Wnt3 pathway (Liu J. et al., 2021). Wu Ping et al. indicated that the expression of the circRNA circux1 was prominently enhanced in hypopharyngeal squamous cell carcinoma (HPSCC) cells and that this circRNA could confer radioresistance to HPSCC through the caspase-1 pathway (Wu et al., 2021). Zhang Guifeng et al. reported that circ-ACAP2 was upregulated in colorectal cancer (CRC) cells and promoted radioresistance via the miR-143-3p/frizzled-4 (FZD4) axis (Zhang G. et al., 2021). CircMTDH.4 was highly expressed in NSCLC cells and can enhance radioresistance through the miR-630/astrocyte elevated gene-1 (AEG-1) axis (Li Y. H. et al., 2020). Moreover, He Yunlong et al. reported that the expression of circVRK1 decreased in ESCC cells and that its overexpression improved tumor radiosensitivity by regulating the miR-624-3p/PTEN/PI3K/AKT signaling pathway (He et al., 2019).

### 3.4 Regulation of other ncRNAs in tumor therapy resistance

In addition to the three classic ncRNAs mentioned above, several other ncRNAs are involved in tumor treatment tolerance, including Piwi-interacting RNAs (piRNAs), small nucleolar RNAs (snoRNAs), and small interfering RNAs (siRNAs), as shown in Supplementary Table S1 and Supplementary Table S2. PiRNAs are a class of ncRNAs with lengths of 24–30 nt that are involved in maintaining the stability of the germline cell genome (Liu et al., 2019). According to previous reports, pi-R17560 is highly expressed in BC cells and may lead to chemotherapy resistance through fat mass- and obesity-associated protein (FTO)-mediated N6-methyladenosine (m6A) demethylation (Ou et al., 2022). Basudeb Das et al. reported that piR-39980 was downregulated in fibrosarcoma cells, where it induced Dox resistance by regulating drug accumulation and DNA repair (Das et al., 2021). SnoRNAs are small RNAs with lengths of 60–300 nucleotides that are present mainly in the nucleolus. They are considered important components of the cellular protein synthesis mechanism (Williams and Farzaneh, 2012). Liu Yonghui et al. indicated that SNORD1C was upregulated in colorectal cancer cells and maintained resistance to 5-FU by activating the Wnt signaling pathway (Liu et al., 2022c). Martina Godel et al. reported that SNORD3A was highly expressed in BC cells and could enhance 5-FU sensitivity in cells by negatively regulating miR-185-5p (Godel et al., 2020). SiRNAs are long double-stranded RNAs 21–23 nucleotides in length that play a role at the posttranscriptional level, especially in RNA interference (RNAi) (Alshaer et al., 2021). It was reported that siRNA targeting glucose transporter protein 1 (GLUT-1) could reduce the radioresistance of laryngeal cancer cells by helping to redistribute the cell cycle phases, reducing DNA repair ability and enhancing apoptosis (Zhong et al., 2019). Moreover, Li Qingjian et al. developed an intracellular pH-responsive nanoparticle (NP) that can transport siRNA and cisplatin targeting Rac-1 into cells to reduce cancer resistance to neoadjuvant chemotherapy (NAC) (Yu et al., 2020).

## 4 Regulatory roles of DDR-related ncRNAs in cancer therapy resistance

As mentioned above, numerous quantities and types of ncRNAs have been found to mediate or regulate the tolerance of cancer cells to radiotherapy and chemotherapy by targeting multiple genes or signaling pathways. Interestingly, several ncRNAs have recently been found to regulate tumor treatment tolerance by altering the DDR pathway, indicating that changes in DDR are crucial for the occurrence and development of tumor resistance, as shown in Table 1. These findings suggest that ncRNAs can also serve as regulatory factors for the DDR to reverse the tolerance of tumor cells, further supporting their potential value as tumor treatment targets.

**TABLE 1:** Regulation of DDR-related ncRNAs in cancer therapy resistance.

ncRNA types	Expression in cancer	Role in cancer	Relationship with DDR	Function in therapy resistance	Related therapies	Related genes or signaling pathways	Cancer types	Refs
MicroRNAs								
miRNA-146a	Upregulated	Oncogene	Pro-DDR	Inducing	Cisplatin	CHOP, LC3-II, DR3, TRB3	Lung cancer	Li Y. H. et al. (2020), He et al. (2021)
miR-33b-3p	Downregulated	Tumor suppressor	Anti-DDR	Inhibiting	Cisplatin	p21WAF1, CIP1, ERCC, NER pathway	Lung cancer	Ou et al. (2015)
miR-1307	Upregulated	Oncogene	Regulated by DDR	Inducing	Folfrinox	CLIC5	Pancreatic cancer	Das et al. (2021)
miR-203	Downregulated	Tumor suppressor	Anti-DDR	Inducing	Oxaliplatin	ATM	Colorectal cancer	Williams and Farzaneh (2012)
miR-211	Downregulated	Tumor suppressor	Anti-DDR	Inhibiting	Carboplatin	TDP1	Ovarian cancer	Liu et al. (2022a)
miR-BART17-5p, miR-BART19-3p	Upregulated	Oncogene	Pro-DDR	Inhibiting	Cisplatin, Doxorubicin	BRCA1, MRN	Nasopharyngeal cancer	Godel et al. (2020)
miR-155	Downregulated	Tumor suppressor	Anti-DDR	Inhibiting	Radiation	RAD51	Breast cancer	Li et al. (2014)
Hsa-let-7	Upregulated	Tumor suppressor	Anti-DDR	Inhibiting	X-ray	ATM, H2AX, Chk1	Gastric cancer	Xu et al. (2016)
miR-18a	Upregulated	Oncogene /Tumor suppressor	Anti-DDR	Inhibiting	Radiation	ATM, H2AX, 53BP1	Breast cancer	Carotenuto et al. (2021)
lncRNAs								
CTBP1-DT	Upregulated	Oncogene	Pro-DDR	Inducing /Inhibiting	Camptothecin	DDUP, RAD18, RAD51C, PCNA, PRRs	Cervical cancer	Chi et al. (2023), Wang et al. (2021a)
ELFN1-AS1	Downregulated	Tumor suppressor	Pro-DDR	Inhibiting	Oxaliplatin	MEIS1	Colorectal cancer	Wang et al. (2019)
lncrna-HOTAIR	Upregulated	Oncogene	Anti-DDR	Inducing	Cisplatin	NF-κB	Ovarian cancer	Lung et al. (2020)
lnc-SBF2-AS1	Upregulated	Oncogene	Anti-DDR	Inducing	Temozolomide		Glioblastoma	Li et al. (2021)
OTUD6B-AS1	Downregulated	Tumor suppressor	Pro-DDR	Inhibiting	Paclitaxel	miR-26a-5p, MTDH	Breast cancer	Alshaer et al. (2021)
LUCAT1	Upregulated	Oncogene	Anti-DDR	Inducing	5-fluorouracil, Camptothecin, Adriamycin Oxaliplatin	PTBP1	Colorectal cancer	Fletcher et al. (2022)
AL133467.2	Upregulated	Oncogene	Anti-DDR	Inducing	Oxaliplatin	ZCCHC4	Hepatocellular carcinoma	Zhou et al. (2020)
NORAD	Upregulated /Downregulated	Oncogene /Tumor suppressor	Anti-DDR /Pro-DDR	Inducing /Inhibiting	Cisplatin Radiation	β-catenin, miR-346, miR-199a-5p	Esophageal squamous cell cancer Prostatic cancer	Zhong et al. (2019), Zhou et al. (2014)
lnc-POP1-1	Upregulated	Oncogene	Anti-DDR	Inducing	Cisplatin	VN1R5	Head and neck squamous cell carcinomas	Brauner et al. (2016)
PDIA3P1	Upregulated	Oncogene	Anti-DDR	Inducing	Doxorubicin	miR125/124, TRAF6	Hepatocellular carcinoma	Shi et al. (2022)
LncPVT1	Upregulated	Oncogene	Anti-DDR	Inducing	Gemcitabine	miR-619-5p, Pygo2	Pancreatic cancer	Yu et al. (2020)
LINC01134	Upregulated	Oncogene	Anti-DDR	Inducing	Radiation	miR-342-3p, IGF2BP2	Hepatocellular carcinoma	Williams et al. (2008)
LncSLCO1C1	Upregulated	Oncogene	Anti-DDR	Inducing	Oxaliplatin	miR-204-5p, miR-211-5p, SSRP1	Gastric cancer	Liu Y. et al. (2021)
MALAT1	Upregulated	Oncogene	Anti-DDR	Inducing	Temozolomide	p50, p53	Glioblastoma	Kristensen et al. (2022)
HOTAIRM	Upregulated	Oncogene	Anti-DDR	Inducing	Cisplatin	EZH2, KU70, KU80, DNA-PKcs, ATM	Breast cancer	Yuan et al. (2020)
lnc-RI	Upregulated	Oncogene	Anti-DDR	Inducing	Radiation	LIG4, NHEJ pathway	Colorectal cancer	Li X. et al. (2020)
SCAT7	Upregulated	Oncogene	Anti-DDR	Inducing	Cisplatin, Camptothecin	TOP1	Lung cancer	Hu et al. (2015)
RBM5-AS1	Upregulated	Oncogene	Anti-DDR	Inducing	Radiation	SIRT6	Medullo blastoma	Song et al. (2011)
CRAL	Upregulated	Oncogene	Anti-DDR	Inducing	Cisplatin	CYLD, AKT	Gastric cancer	Maekawa et al. (2018)
DNM3OS	Upregulated	Oncogene	Anti-DDR	Inducing	Radiation	CAFs	Esophageal squamous cell cancer	Dai et al. (2020)
CRNDE	Upregulated	Oncogene	Anti-DDR	Inducing	Radiation	SP1, PDK1	Hepatocellular carcinoma	Zhang G. et al. (2021)
linc00312	Downregulated	Tumor suppressor	Pro-DDR	Inhibiting	Radiation	DNA-PKcs, Ku80	Nasopharyngeal carcinoma	Sun et al. (2021)
lnc-TALC	Upregulated	Oncogene	Anti-DDR	Inducing	Temozolomide	M2 macrophage polarization	Glioblastoma	Li Y. et al. (2022)
circRNAs								
circ-AKT3	Upregulated	Tumor suppressor	Pro-DDR	Reducing	Cisplatin	miR-198, PIK3R1	Gastric cancer	Qian et al. (2020)
circ-MTHFD1L	Upregulated	Oncogene	Pro-DDR	Inducing	Gemcitabine	HR pathway	Pancreatic ductal adenocarcinoma	Lal et al. (2023)
CircRNA-001895	Upregulated	Oncogene	Pro-DDR	Inducing	Sunitinib	γH2AX, p-DNA-PKcs	Renal cell carcinoma	Huan et al. (2020)
circSMARCA5	Downregulated	Oncogene	Pro-DDR	Inducing	Drugs (NM)	SMARCA5	Breast cancer	Zhu et al. (2022)
circNEIL3	Upregulated	Tumor suppressor	Anti-DDR	Inducing	Radiation	miR-1184, PIF1	Lung cancer	Liu P. et al. (2021)
circ-NEK6	Upregulated	Tumor suppressor	Anti-DDR	Reducing	Iodine 131I	miR-370-3p, MYH9	Thyroid cancer	Yu et al. (2022)
Others								
piR-39980	Upregulated	Tumor suppressor	Anti-DDR	Reducing	Doxorubicin	RRM2, CYP1A2	Fibrosarcoma	Chen Y. et al. (2021)
SNORD3A, SNORA13, SNORA28	Upregulated	oncogene	Pro-DDR	Inducing	Doxorubicin	GADD45A, MYC, TOP2A	Osteosarcoma	Peng et al. (2020)
RRP9	Upregulated	oncogene	Pro-DDR	Inducing	Gemcitabine	IGF2BP1, AKT pathway	Pancreatic cancer	Statello et al. (2021)
siMGMT	Upregulated	Tumor suppressor	Anti-DDR	Reducing	Trimetazidine	MGMT	Glioma	Zhu et al. (2021)
siATM siDNA-PKcs	Upregulated	Tumor suppressor	Anti-DDR	Reducing	Alkylating agent Radiation	ATM, DNA-PKcs	Prostatic cancer	Guo et al. (2021)

NM, Not mentioned

### 4.1 Regulation of DDR-related miRNAs in cancer therapy resistance

#### 4.1.1 DDR-related miRNAs in cancer chemotherapy resistance

**FIGURE 3:**
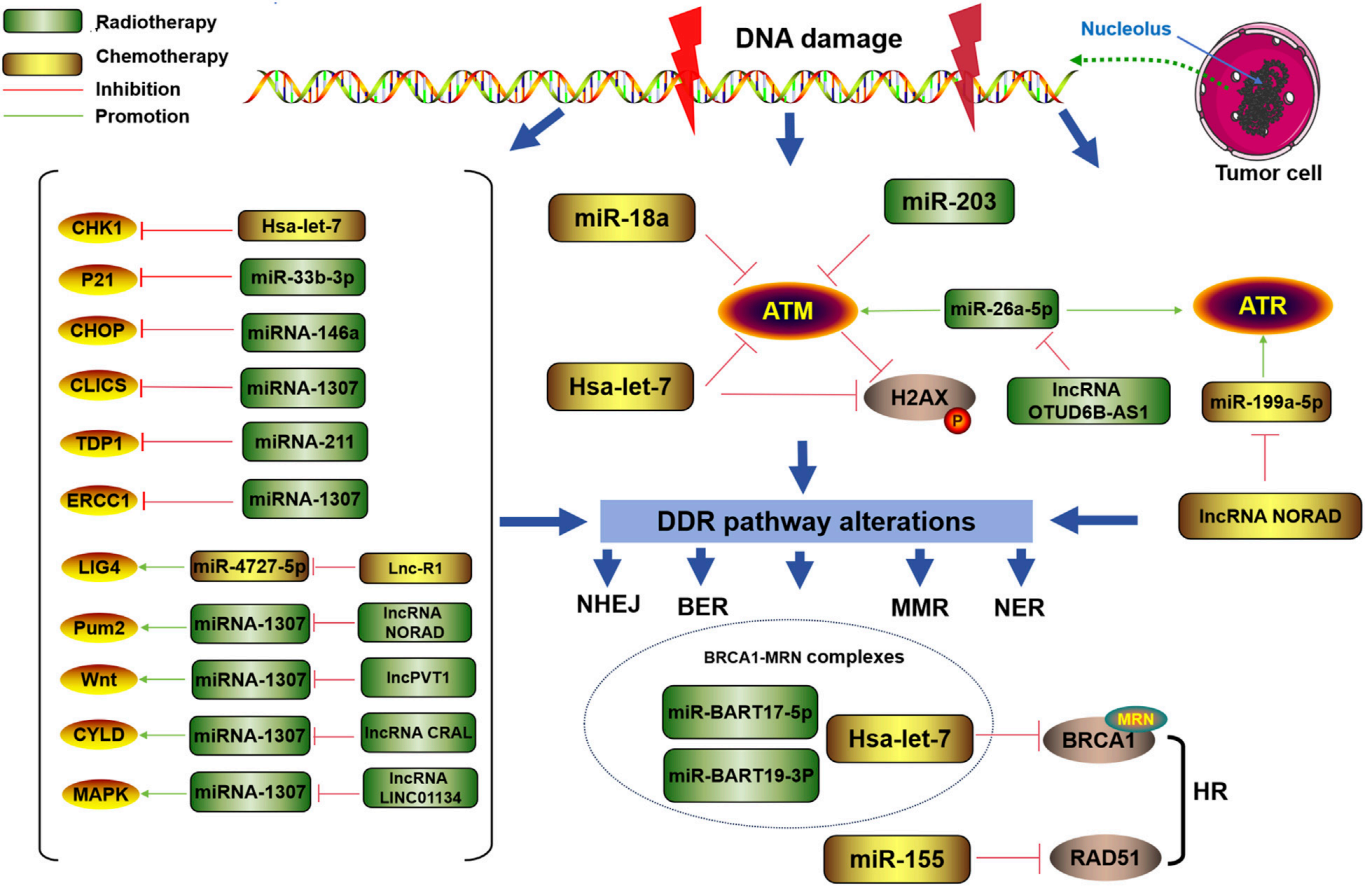
Regulatory role of DDR-related miRNAs in tumor chemotherapy or radiotherapy resistance DDR-related miRNAs can mediate changes in the DDR pathway to regulate radiation or chemotherapy resistance in tumor cells.

As shown in Figure 3, DDR-related miRNAs can strongly influence cancer chemotherapy tolerance. Tan W et al. reported that the upregulation of miRNA-146a increased resistance to chemotherapy (CDDP) by targeting the degradation of DNA damage-inducible transcript 3 (DDIT3) in lung cancer cells (Maekawa et al., 2018). It has long been known that the upregulation of miRNA-146a is closely related to the occurrence of lung cancer (Williams et al., 2008). Recently, it was further discovered that miRNA-146a can alter the chemotherapy sensitivity of cancer cells by downregulating C/EBP homologous protein (CHOP), leading to a decreased expression levels of light chain 3-II (LC3-II), death receptor 5 (DR5), and tribble homolog 3 (TRB3), further affecting the progression of lung cancer (Li et al., 2014). Xu S et al. reported that miR-33b-3p facilitates the DDR against cisplatin treatment in lung cancer cells by targeting p21^WAF1/CIP1^. Furthermore, they reported that ectopic expression of miR-33b-3p increased the expression of excision repair cross-complementation group 1 (ERCC1), which is crucial to the NER pathway for cisplatin-DDR (Xu et al., 2016). Carotenuto P et al. reported that an increase in the expression of MIR1307 can induce a reduction in chloride intracellular channel 5 (CLIC5) expression, resulting in protection against DNA damage caused by FOLFIRINOX (a combination of the chemical drugs fluorouracil, oxaliplatin, and irinotecan-FOI), ultimately leading to FOLFIRINOX resistance (Carotenuto et al., 2021). Zhou Y et al. reported that through inhibiting ATM kinase, miR-203 causes oxaliplatin resistance in CRC. During this process, cancer cells gain an improved capacity to repair damaged DNA or to decrease the activation of the DNA damage response system to prevent apoptosis (Zhou et al., 2014). Wang T et al. noted that miR-211 overexpression can promote platinum sensitivity in ovarian cancer by inhibiting the expression of the TDP1 gene and certain DDR genes, thereby affecting the prognosis of the disease (Wang et al., 2019). Lung RW et al. reported that Epstein–Barr virus (EBV)-encoded miRNAs (miR-BARTs, especially miR-BART17-5p and miR-BART19-3p) can reduce DDR ability by targeting BRCA1, increasing the sensitivity of nasopharyngeal carcinoma cells to cisplatin and doxorubicin (Lung et al., 2020).

In addition, other types of ncRNAs can target miRNAs and regulate the chemotherapeutic tolerance of tumor cells via the DDR. Li PP et al. reported that overexpression of the lncRNA OTUD6B-AS1 increased paclitaxel resistance in triple-negative breast cancer patients through the downregulation of miR-26a-5p and the overexpression of metadherin (MTDH). The OTUD6B-AS1/miR-26a-5p/MTDH axis can inhibit DDR by blocking the phosphorylation and activation of RAD51, ATR, and ATM (Li et al., 2021). Fletcher CE et al. reported that the genomic protective lncRNA NORAD interacts with miR-346, blocking the association between pumilio RNA-binding family member 2 (Pum2) and miR-346 and increasing the turnover of DDR transcripts, thereby inducing the tolerance of prostate cancer to PARPis (Fletcher et al., 2022). The competitive binding of lncPVT1 with miR-619-5p regulates the target genes Pygopus 2 (PYGO2) and autophagy-related gene 14 (ATG14) of miR-619-5p to activate the Wnt/β-catenin pathway, increasing the chemoresistance of pancreatic cancer to gemcitabine (Zhou et al., 2020). The lncRNA CRAL has also been reported to enhance cisplatin resistance in GC cells by absorbing miR-505 and upregulating CYLD lysine 63 deubiquitinase (CYLD) expression (Chi et al., 2023). Interestingly, the lncRNA LINC01134 can promote the activation of miR-342-3p and insulin-like growth factor 2 (IGF2) mRNA-binding protein 2 (IGF2BP2), thereby activating the MAPK pathway to promote the repair of DNA damage and increase chemotherapy resistance in HCC (Wang et al., 2021b).

#### 4.1.2 Regulation of DDR-related miRNAs in cancer radioresistance

DDR-related miRNAs play crucial roles in modulating cancer cell response to radiotherapy, influencing therapeutic efficacy through various molecular mechanisms (Figure 1). According to Gasparini et al., in triple-negative breast cancer cells with high expression of miR-155, homologous recombination and subsequent DNA damage repair after radiation therapy are reduced, increasing sensitivity to radiation. Furthermore, miR-155 can inhibit the recombinant enzyme RAD51 in cancer to control DNA repair activity and γ-radiation (IR) sensitivity (Gasparini et al., 2014). Hu H et al. reported that the miRNA Hsa let-7g indirectly inhibited DDR-related genes and increased the sensitivity of GC to treatment-induced oxidative stress (Hu et al., 2015). Song L et al. reported that ectopic expression of miR-18a inhibited IR-induced DDR and reduced the frequency of HRR, thereby increasing the radiosensitivity of breast cancer cells (Song et al., 2011).

In addition, miRNAs can also be targeted and regulated by lncRNAs to alter the radioresistance of tumor cells via the DDR. For example, the lncRNA NORAD is involved in inhibiting the expression of miR-199a-5p and the ATR/Chk1 pathway, ultimately weakening the effect of anti-PD-1 inhibitors and increasing radiation resistance in ESCC (Sun et al., 2021). Lnc-RI can competitively bind miR-4727-5p to promote the expression of DNA ligase IV (LIG4), thereby enhancing the NHEJ pathway or inducing decreased tumor cell apoptosis, leading to reduced radiation sensitivity in CRC cells (Li X. et al., 2020).

### 4.2 Regulation of DDR-related lncRNAs in cancer therapy resistance

**FIGURE 4:**
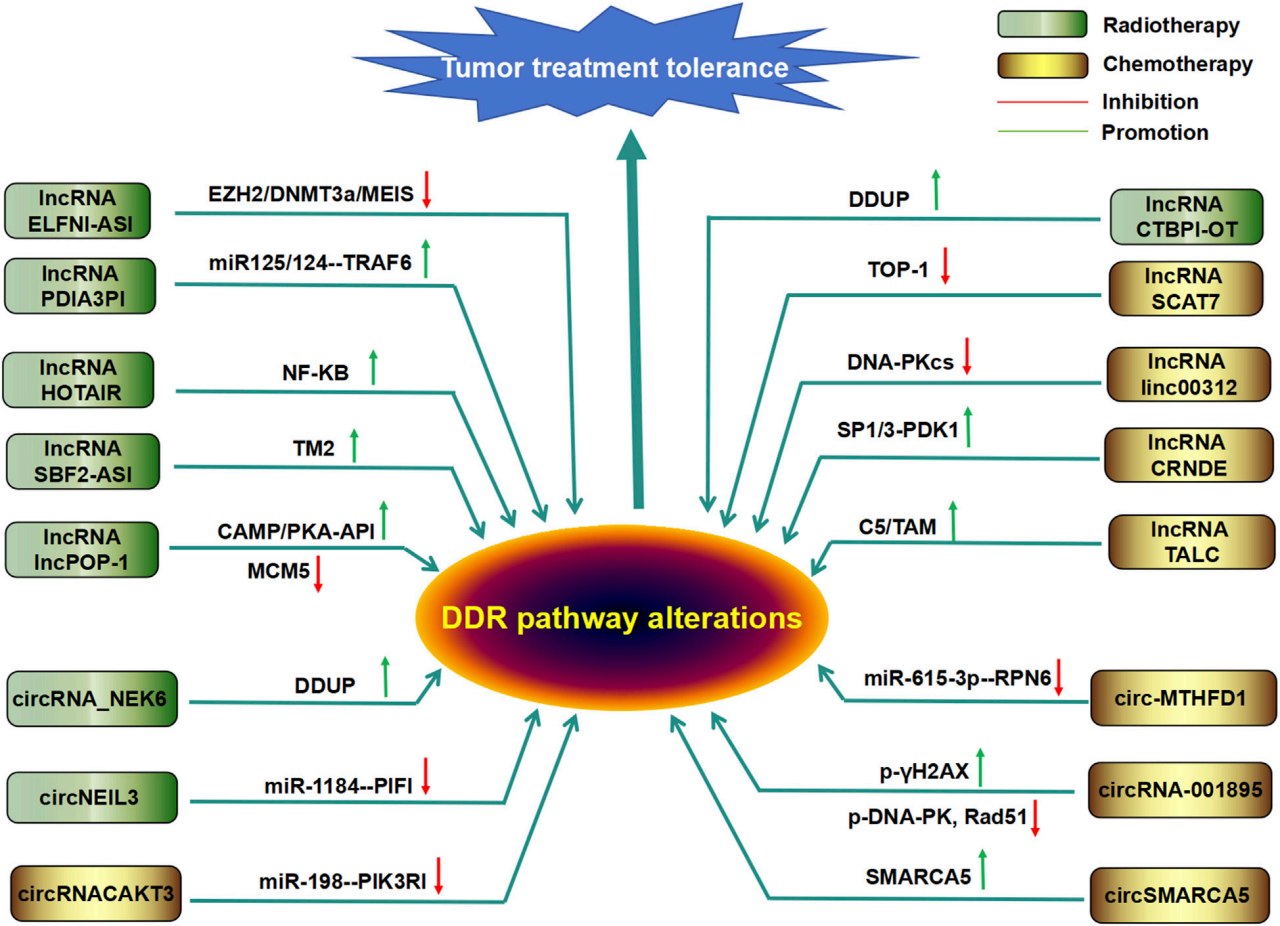
Regulatory roles of DDR-related lncRNAs and circRNAs in tumor chemotherapy or radiotherapy resistance Many DDR-related lncRNAs and circRNAs mediate or enhance tumor radiotherapy or chemotherapy.

Research has shown that abnormal expression of lncRNAs in cancer cells can reduce sensitivity to chemotherapy or radiotherapy by activating or inhibiting the DDR pathway. Chemotherapy drugs and radiation-mediated DNA damage can also induce changes in the expression of lncRNAs in tumor cells, thus regulating the DDR pathway and affecting cell tolerance, as shown in Figure 4. These data indicate that the overexpression or inhibition of these DDR-related lncRNAs can be an effective means of cancer treatment.

#### 4.2.1 DDR-related lncRNAs in cancer chemotherapy tolerance

According to reports, the integration site 1 (MEIS1) of neutrophil eosinophilic leukemia can damage the vitality of mouse CRC cells and tumor growth and enhance the sensitivity of CRC cells to oxaliplatin by preventing DNA damage repair (Zhang M. et al., 2021). However, in CRC, MEIS1 expression is significantly reduced and inversely proportional to patient survival. Further research has shown that lncRNA ELFN1–AS1 is localized in the promoter of MEIS1 by interacting with EZH2–DNA methyltransferase 3A (DNMT3A) to inhibit MEIS1 translation (Li Y. et al., 2022), suggesting that ELFN1 antisense RNA 1 (ELFN1-AS1) induces sensitivity to oxaliplatin in colon cancer cells through the EZH2/DNMT3a/MEIS1 axis. Upregulation of the lncRNA HOTAIR can induce nuclear factor (NF)-κB in ovarian cancer. Its prolonged activation or expression causes cells to enter a sustained state of maintaining the DDR to increase tumor cell resistance to CDDP chemotherapy (Qian et al., 2020). Similarly, lnc-SBF2-AS1 is highly expressed in glioblastoma and can enhance cell damage repair to DSBs, leading to tumor cells being chemically resistant to TMZ (Lal et al., 2023). In addition, some lncRNAs can bind to DDR-related molecules to regulate DDR in cancer. For example, the lncRNA LUCAT1 can bind to polypyrimidine tract-binding protein 1 (PTBP1), which is associated with DNA damage genes and contributes to chemotherapy tolerance in colorectal cancer cells (Huan et al., 2020). lncRNA-AL133467.2 inhibits DNA damage induced HCC cell apoptosis by interacting with zinc finger CCHC domain-containing protein 4 (ZCCHC4), thereby mediating tumor cell resistance to DNA damage agents (DDA) (Zhu et al., 2022). LncSLCO1C1 can interact with scaffold-based structure-specific recognition protein 1 (SSRP1)/H2A/H2B complexes and competitively bind with miR-211-5p and miR-204-5p to increase SSRP1 expression, enhance cell growth, prevent DNA damage, and induce chemotherapy drug tolerance in gastric cancer cells (Liu Y. et al., 2021). Of course, in addition to lncSLCO1C1, as mentioned above, there are also some lncRNAs that can mediate tumor cell tolerance to chemical drugs by regulating miRNAs and their target genes, including lncRNAOTUD6B-AS1, NORAD, lncPVT1, CRAL, and LINC01134 (Jackson and Bartek, 2009; Shao et al., 2019; Wang et al., 2020; Wang et al., 2021a; Wu et al., 2021).

The lncRNA CTBP1-DT is highly expressed in cancer cells, acts as an oncogene, and is further regulated by the cellular environment (Liu P. et al., 2021). Interestingly, camptothecin (CPT) was reported to not affect the expression of the lncRNA CTBP1-DT but can significantly induce the expression of the lncRNA CTBP1-DT, which encodes the microprotein DNA damage-upregulated protein (DDUP) (Yu et al., 2022). Furthermore, CPT-induced DNA damage-mediated phosphorylation of DDUP results in a drastic “dense to loose” conformational change in DDUP structure, maintaining the retention of radiation-sensitive 18 (RAD18) at the site of DNA damage, thereby promoting DNA damage repair through dependent RAD51C-mediated HRR and single ubiquitination proliferating cell nuclear antigen (PCNA)-mediated pattern recognition receptor (PRR) mechanisms (Yu et al., 2022). Interestingly, treatment with the ATR inhibitor berzoserib can significantly inhibit the formation of DDUP lesions, leading to the rapid elimination of RAD18-and PCNA-expressing lesions and ultimately to the hypersensitivity of ovarian cancer cells to DNA destructive chemotherapy drugs (Yu et al., 2022). The lncRNA lnc-POP1-1 is induced by vomeronal type 1 receptor 5 (VN1R5), a protein whose expression is significantly increased in cisplatin-resistant HNSCC cells and tissues, through the activation of specific protein 1 (Sp1) transcription factors via the cyclic AMP (cAMP)/protein kinase A (PKA) pathway. Additionally, lnc-POP1-1 directly binds to the microsomal maintenance deficient type 5 (MCM5) protein and slows the degradation of MCM5 by inhibiting its ubiquitination, thereby promoting the repair of DNA damage caused by cisplatin (Brauner et al., 2016). The lncRNA PDIA3P1 can be upregulated by silencing hMTR4, which assists tumors in preventing apoptosis caused by Dox (Xie et al., 2020). Mechanistically, PDIA3P1 combines with the miR-125/124 pathway to promote the expression of TRAF6, which can increase chemoresistance (Xie et al., 2020). In addition, the expression of metastasis-associated lung adenocarcinoma transcript 1 (MALAT1) was increased in a manner dependent on nuclear factor-κB (NF-κB) and p53 in GBM cells treated with temozolomide (Kristensen et al., 2022). The depletion of MALAT1 significantly increased the cytotoxicity of GBM cells to temozolomide, while the *in vivo* application of anti-MALAT1 siRNA increased the efficacy of temozolomide in glioma xenograft mice (Kristensen et al., 2022).

#### 4.2.2 DDR-related lncRNAs in cancer radiotherapy resistance

The lncRNA HOTAIR is highly expressed in cancer cells and promotes the expression of DDR factors, leading to radioresistance in breast cancer (Chen Y. et al., 2021). The DNA damage-induced lncRNA SCAT7 targets the degradation of topoisomerase I (TOP1), induces the activation and maintenance of DDR, and leads to chemotherapy resistance of lung adenocarcinoma to cisplatin and camptothecin (Statello et al., 2021). Cancer-associated fibroblasts (CAF) can enhance the synthesis of lncRNA dynamin three opposite strand (DNM3OS) in a platelet-derived growth factor-β (PDGFβ/platelet-derived growth factor receptor β(PDGFRβ)/Forkhead box transcription factor O1 (FOXO1) signaling pathway-dependent manner, leading to the DDR and increased radiosensitivity in ESCC (Zhang H. et al., 2019). The interaction between lncRNA RBM5-AS1 and SIRT6 can also induce the DDR to enhance radiation resistance in medulloblastoma (Zhu et al., 2021). lncRNA CRNDE has also been reported to participate in radiation resistance by modulating the SP1/3-phosphoinositide-dependent protein kinase 1 (PDK1) axis in hepatocellular carcinoma (Zhang G. et al., 2021). In contrast, overexpression of lncRNA linc00312 in nasopharyngeal carcinoma can inhibit the expression of DNA-dependent protein kinase catalytic subunit (DNA-PKcs), thereby reducing their binding to the Ku80 protein, inhibiting DNA damage repair, and reducing the radiation resistance of tumor cells (Guo et al., 2021).

In addition, the lncRNA TMZ-related lncRNA (lnc-TALC) in cases of GBM recurrence can be secreted into extracellular vesicles and transmitted to tumor-associated macrophages to promote the M2 polarization of microglia (Li Y. et al., 2022). The M2 polarization of microglia can induce the secretion of complement component C5/C5a, promote the phosphorylation of p38 MAPK and the repair of DNA damage induced by TMZ, and lead to chemotherapy resistance (Li Y. et al., 2022). This suggests that lnc-TALC transported by extracellular vesicles can reshape the GBM microenvironment and reduce the sensitivity of tumors to TMZ chemotherapy.

### 4.3 Regulation of cancer therapy resistance by DDR-related circRNAs

Compared to those of the other two types of ncRNAs, there are currently fewer circRNAs found in tumors related to chemotherapy or radiotherapy tolerance.

#### 4.3.1 DDR-related circRNAs in cancer chemotherapy resistance

CircAKT3 (hsa_circ_0000199, a circRNA originating from exons 8, 9, 10, and 11 of the AKT3 gene) was upregulated in CDDP-resistant GC tissues and cells compared with CDDP-sensitive samples (Huang et al., 2019). Further *in vivo* and *in vitro* experiments have confirmed that circAKT3 promotes DNA damage repair and inhibits apoptosis by absorbing miR-198 to promote the expression of PI3 kinase p85 alpha (PIK3R1) in GC cells (Huang et al., 2019). Hsa_circ_0078297 (circ-MTHFD1L) was also significantly increased in pancreatic ductal adenocarcinoma (PDAC) tissues and cells (Chen Z. et al., 2022). Circ-MTHFD1L can also upregulate the expression of the ribophorin family 6 (RPN6), an endogenous miR-615-3p molecular sponge, thereby promoting the HR pathway for DNA damage repair and enhancing the chemotherapeutic resistance of tumor cells to gemcitabine. However, the combination of silent circ-MTHFD1L and olaparib can increase the sensitivity of cancer to gemcitabine (Chen Z. et al., 2022). Similarly, circRNA-001895 was significantly upregulated in sunitinib-resistant renal cell carcinoma (RCC) tissues and cells (Tan et al., 2023). Knockout of circRNA_001895 in sunitinib-resistant RCC cells inhibited cell proliferation and promoted cell apoptosis by inducing the phosphorylation of the histone H2AX (γH2AX) and reducing phosphorylation of DNA-dependent protein kinase (p-DNA-PK) and Rad51 (Tan et al., 2023). In addition, in contrast to that in the host gene SMARCA5 (an ATPase of the ISWI class of chromatin remodelers), the expression level of circSMARCA5 in breast cancer tissue is significantly reduced, and circSMARCA5 can bind to its parent locus to form an R ring, which leads to the suspension of the transcription of exon 15 of SMARCA5 (Dogan et al., 2023). Overexpression of circSMARCA5 can induce drug sensitivity in breast cancer cell lines by inhibiting the expression of SMARCA5 and the production of truncated nonfunctional proteins (Dogan et al., 2023). These data suggest that circRNAs may serve as therapeutic targets for treating drug resistance.

#### 4.3.2 DDR-related circRNAs in cancer radioresistance

CircNEIL3 is a significantly downregulated circRNA in lung adenocarcinoma (LUAD) cells treated with 0, 2, or 4 Gy of radiation (Zhang T. et al., 2022). Overexpression of circNEIL3 can significantly inhibit radiation-induced cell ptosis by regulating the miR-1184/phytochrome-interacting factor 1 (PIF1) axis and triggering the activation of absent in melanoma 2 (AIM2) inflammasomes, inducing tumor cell tolerance to radiation therapy (Zhang T. et al., 2022). The expression of circ-NEK6 was significantly increased in iodine-131 (131I)-resistant differentiated thyroid cancer (DTC) tissues and cell lines (Chen F. et al., 2021). Silencing of circ-NEK6 in iodine 131I-resistant cells inhibited cell proliferation, migration, and invasion while inducing cell apoptosis and DNA damage by regulating the miR-370-3p/nonmuscle myosin heavy chain IIA (MYH9) axis (Yu et al., 2022). These results indicate that CircNEIL3 and circ_NIMA-related kinase-6 (NEK6) may be potential biomarkers and therapeutic targets for cancer patients with radiation resistance.

### 4.4 Other DDR-related ncRNAs regulate cancer therapy resistance

There are also some other types of DDR-related ncRNAs involved in tumor chemotherapy drug tolerance or radiotherapy tolerance, including piRNAs, snoRNAs, and siRNAs (Table 1). For example, the overexpression of piR-39980 not only significantly inhibits the proliferation, migration, ROS generation, and colony formation of tongue squamous cell carcinoma (TSCC) cells but also inhibits the expression of farnesyl-diphosphate farnesyltransferase 1 (FDFT1). Moreover, the inhibition of FDFT1 induces hypoxia, which slows DNA repair and the accumulation of damaged DNA, leading to tumor cell death (Chattopadhyay and Mallick, 2023). piR-39980 can decrease fibrosarcoma chemoresistance to DOX by regulating ribonucleotide reductase subunit M2 (RRM2) and cytochrome P450 enzyme 1A2 (CYP1A2), indicating that piRNAs can bind to DOX for cancer treatment (Das et al., 2021). A group of upregulated snoRNAs, particularly SNORD3A, SNORA13, and SNORA28, have been detected in human anti-Dox osteosarcoma cells (Godel et al., 2020). Overexpression of these three snoRNAs can significantly reduce the cytotoxicity of Dox, induce upregulation of growth arrest and DNA damage protein 45A (GADD45A) and MYC, and downregulate type II topoisomerase A (TOP2A). These findings suggest that snoRNAs induce tumor cell resistance to Dox by regulating the expression of genes involved in DNA damage perception, DNA repair, ribosomal biogenesis, and proliferation (Godel et al., 2020). Ribosomal RNA processing 9 (RRP9) is an evolutionarily conserved U3-snoRNP protein that is crucial for early preribosomal RNA (rRNA) processing and cleavage and is expressed at higher levels in pancreatic cancer tissues than in normal tissues (Zhang Z. et al., 2022). RRP9 has been shown to activate the AKT signaling pathway by interacting with the deoxyribonucleic acid-binding region of IGF2 mRNA-binding protein 1 (IGF2BP1) *in vivo* and *in vitro*, thereby promoting the resistance of prostate cancer cells to gemcitabine (Zhang Z. et al., 2022). Hypoxia radiation-sensitive nanoparticles (RDPP (Met)/TMZ/siMGMT) that can effectively release TMZ and small interfering O6 methylguanine DNA methyltransferase RNA (siMGMT) have been reported to effectively penetrate the blood‒brain barrier, accurately target glioma cells, and inhibit cell proliferation (Xie et al., 2022). These findings suggest that RDPP (Met)/TMZ/siMGMT can effectively improve the chemotherapeutic efficacy and radiation sensitivity of temozolomide in glioma (Xie et al., 2022). In addition, siRNA-mediated ATR silencing promoted the sensitivity of cancer cells to methyl methanesulfonate (MMS) (Collis et al., 2003), further supporting the enormous potential of using siRNAs in tumor treatment.

## 5 Conclusions, perspectives and discussions

In summary, many recent studies have shown that ncRNAs (mainly miRNAs, lncRNAs and circRNAs) play important regulatory roles in tumor chemotherapy and radiation tolerance by targeting changes in the DDR. These ncRNAs either activate or maintain the DDR to induce therapeutic tolerance in tumor cells or block the DDR to induce cell apoptosis and enhance the cytotoxicity of chemotherapy drugs or radiotherapy. Cancer has become the second leading cause of death worldwide due to its rapid metastasis, spread, recurrence, and other factors (Xu et al., 2016). Therefore, cancer is the main focus of ongoing worldwide research in biomedicine. Radiotherapy and chemotherapy are important methods for treating malignant tumors, and tumor tolerance is a key factor influencing the effectiveness of tumor treatment. Recent observations based on increasing evidence suggest that ncRNAs may affect DNA damage repair and play important regulatory roles in tumor radiotherapy and chemotherapy tolerance (Volpato et al., 2007). Moreover, DDR-related ncRNAs may be targets for estimating cancer treatment responses, thus contributing to the development of treatment designs targeting unique individuals. Many ncRNAs have been proven to be important molecules that affect the occurrence and development of tumors. Indeed, with the deepening of clinical research, a new ncRNA scoring system has emerged that combines PD-L1 expression, tumor change load, and cytotoxic T lymphocyte (CTL) infiltration for precise tumor immunotherapy (Zhong et al., 2019). In addition, a new biomarker, extracellular ncRNAs, has emerged for clinical diagnosis. As a liquid biopsy method, these extracellular ncRNAs can be used to predict and diagnose the recovery of physiological and pathological states after treatment with platinum-containing neoadjuvant chemotherapy (Thomsen and Vitetta, 2018). This is partly due to their stability in human body fluids and peripheral blood, as well as their specific expression in diseases. For example, certain miRNAs (such as miR-136 and upregulated miR-27b) exhibit differential expression between healthy subjects and oral cancer patients (Williams et al., 2008; Baraniskin and Schroers, 2021). Similarly, the high expression levels of many lncRNAs, such as LINC00665, can distinguish between diseased breast cells/cancer tissues and normal tissues, indicating the potential diagnostic value of lncRNAs in tumors (Godel et al., 2020). In addition, due to the positive correlations between the expression of certain ncRNAs and clinical stage, metastasis, and patient survival, ncRNAs can be considered prognostic molecular markers for oral cancer and other cancers (Crooke et al., 2018). Therefore, the DDR-related ncRNAs mentioned in this article may be ideal biomarkers for cancer prognosis and recurrence diagnosis.

Translating miRNA-based strategies into clinical applications for cancer treatment presents significant challenges that need to be addressed. These challenges include effectively delivering miRNAs to target cells, minimizing off-target effects, managing immune responses, optimizing dosing, and conducting rigorous clinical validation. However, along with these challenges come promising opportunities. miRNA-based therapies have the potential for precision medicine by allowing tailored treatments based on individual tumor profiles. They can also be integrated with existing therapies to enhance efficacy and overcome resistance. Additionally, certain miRNAs can serve as biomarkers for diagnosing and predicting prognosis in cancer patients. The diverse regulatory roles of miRNAs in oncogenic pathways provide numerous therapeutic targets. Advancements in delivery technologies such as nanoparticles and viral vectors are improving the specificity and efficiency of miRNA delivery. Furthermore, evolving regulatory frameworks support the translation of ncRNA-based therapeutics into clinical practice. Addressing these challenges while capitalizing on these opportunities is crucial for realizing the transformative potential of miRNA-based strategies in improving cancer treatment outcomes.

Moreover, with the development of biotechnology, such as high-throughput sequencing, functional research on ncRNAs related to the DDR could provide new prospects for cancer treatment. Numerous studies have elucidated the relationship between DDR-related ncRNAs and tumor treatment tolerance. Therefore, DDR-related ncRNAs are currently among the most interesting topics for researchers in anticancer therapy, with the goal of thoroughly understanding the pathways that regulate and control their molecular mechanisms. A large amount of evidence suggests that tumor cells typically exhibit altered DNA damage repair ability, and the underlying mechanisms are being increasingly studied. However, the mechanisms of action of these DDR-related ncRNAs involve multiple complex factors, and the current review clarifies only the tip of the iceberg by combining previous literature and research results. However, further studies are needed to determine the optimal detection methods for ncRNAs related to the DDR in patients, their diagnostic value for cancer recurrence, and the development of personalized therapies. In addition, further research is needed to evaluate the effectiveness of DDR-related ncRNA drugs in clinical settings. Overall, a comprehensive understanding of the important role of ncRNAs in tumor treatment tolerance will be beneficial for improving the rational use of drugs and the development of new drugs.

## Figures and Tables

**Table:** 

**ADP-ribose**	Inhibitors of poly
**AEG-1**	Astrocyte elevated gene-1
**AIM2**	Absent in melanoma 2
**AKT**	Protein kinase
**AP**	Apurinic/apyrimidinic
**APE1**	AP endonuclease 1
**APTX**	Apatasin
**ASF1B**	Anti-silencing function 1B
**ATG5**	Autophagy-related gene 5
**ATG14**	Autophagy-related gene 14
**ATM**	Ataxia telangiectasia mutated
**ATR**	Ataxia-telangiectasia-mutated-and-Rad3-related kinase
**BC**	Breast cancer
**BER**	Base excision repair
**BRCA1**	BReast-CAncer susceptibility gene 1
**CAF**	Cancer-associated fibroblasts
**cAMP**	Cyclic AMP
**CASP9**	Caspase-9
**CD**	Differentiation cluster
**CDDP**	Cisplatin
**Cdc25A**	Cell division cycle 25 A
**CDKs**	Cyclin-dependent kinases
**CHK1**	Checkpoint kinase 1
**CHK2**	Checkpoint kinase 2
**CHOP**	C/EBP Homologous Protein
**cGAS-STING**	Cyclic GMP AMP synthase stimulator
**circRNAs**	Circular RNAs
**CLIC5**	Chloride intracellular channel 5
**CPT**	Camptothecin
**CRC**	Colorectal cancer
**CS**	Cockayne syndrome
**CTL**	Cytotoxic T lymphocytes
**CYLD**	CYLD lysine 63 deubiquitinase
**CYP1A2**	Cytochrome P450 enzyme 1A2
**DDA**	DNA damage agents
**DDIT3**	DNA damage inducible transcript 3
**DDR**	DNA damage repair or DNA damage response
**DDRi**	DDR inhibitors
**DDUP**	DNA damage-upregulated protein
**DNA-PK**	DNA-dependent protein kinase
**DNA-PKcs**	DNA-dependent protein kinase catalytic subunit
**DNMT3A**	DNA methyltransferase 3A
**DNM3OS**	Dynamin 3 Opposite Strand
**DOX**	Doxorubicin
**DR5**	Death receptor 5
**DSBR**	Double strand break repair
**DSBs**	DNA double strand breaks
**DTC**	Differentiated thyroid cancer
**EBV**	Epstein-Barr virus
**EIF5A2**	Eukaryotic initiation factor-5A2
**EGFR**	Epidermal growth factor receptor
**ELFN1-AS**	ELFN1 antisense RNA 1
**ERCC1**	Excision repair cross-complementation group 1
**ESCC**	Esophageal squamous cell carcinoma
**EZH2**	Enhancer of zeste homolog 2
**FAT**	Frap-atm-trap
**FDFT1**	Farnesyl-Diphosphate Farnesyltransferase 1
**FEN1**	Flap endonuclease 1
**FOLFIRINOX**	A combination of chemotherapy drugs Fluorouracil, Oxaliplatin, Irinotecan-FOI
**FOS**	c-FOS
**FOXO1**	Forkhead box transcription factor O1
**FTO**	Fat mass and obesity-associated protein
**FZD4**	Frizzled-4
**GADD45A**	Growth arrest and DNA damage protein 45A
**GBM**	Glioblastoma
**GC**	Gastric cancer
**GG-NER**	Global Genome NER
**GLUT-1**	Glucose transporter protein 1
**HCC**	Hepatocellular carcinoma
**HIF-1α**	Hypoxia-inducible factor 1
**hMTR4**	Homologue of mRNA transport mutant 4
**HNSCC**	Head and neck squamous cell carcinoma
**HPSCC**	Hypopharyngeal squamous cell carcinoma
**H2A/H2B**	Nucleosomal core histones
**HRR**	Homologous recombination repair
**IGF2**	Insulin-like growth factor 2
**IGF2BP**	IGF2 mRNA binding proteins
**IR**	γ Radiation
**LC3-II**	Light chain 3-II
**LIG4**	DNA Ligase IV
**lncRNAs**	Long non coding RNAs
**LUAD**	Lung adenocarcinoma
**MAPK**	Mitogen-activated protein kinase
**MALAT1**	Metastasis-associated lung adenocarcinoma transcript 1
**Mcl-1**	Myeloid cell leukemia 1
**MCM5**	Maintenance deficient type 5
**miRNAs**	MicroRNAs
**MEF2D**	Myocyte enhancer factor 2D
**MEIS1**	Integration site 1
**MEK**	Mitogen-activated protein kinase
**MET**	Mesenchymal-epithelial transition factor
**MGMT**	Methylguanine-DNA methyltransferase
**MMR**	Mismatch repair
**MMS**	Methyl methanesulfonate
**MSI**	Microsatellite instability
**MTDH**	Metadherin
**ncRNAs**	Non coding RNAs
**MutLα**	Heterodimer of mutL homologue 1 (MLH1) and postmeiotic segregation increased 2 (PMS) proteins
**MYC**	c-MYC
**MYH9**	Nonmuscle myosin heavy chain IIA
**m6A**	N6-methyladenosine
**NAC**	Neoadjuvant chemotherapy
**NEDD4L**	4-like protein
**NEK6**	NIMA-related kinase-6
**NER**	Nucleotide excision repair
**NF**	Nuclear factor
**NF-κB**	Nuclear factor-κB
**NHEJ**	Non homologous terminal junction
**NP**	Nanoparticle
**Nrf2**	Nuclear factor-erythroid 2-related factor 2
**NSCLC**	Non-small cell lung cancer
**OGG1**	8-oxoguanine-DNA glycosylase
**PARP**	Polymerase
**PARPi**	Polymerase inhibitors
**PCNA**	Proliferating cell nuclear antigen
**PDAC**	Pancreatic ductal adenocarcinoma
**PDGF β**	Platelet-derived growth factor β
**PDGFR β**	Platelet-derived growth factor receptor
**PDIA3P1**	Protein disulfide isomerase family A member 3 pseudogene 1
**PDK1**	3-phosphoinositide-dependent protein kinase 1
**PD-L1**	Programmed death ligand 1
**p-DNA-PK**	Phosphorylated DNA dependent protein kinase
**PIF1**	Phytochrome-interacting factor1
**PIKK**	Phosphatidylinositol 3-kinase-like protein kinase
**PIK3AP1**	Phosphoinositol-3-kinase adaptor protein 1
**PIK3R1**	PI 3 Kinase p85 alpha
**piRNAs**	Piwi-interacting RNA
**PI3K**	Phosphatidylinositol-3 kinase
**PKA**	Protein kinase A
**PNKP**	Polynucleotide kinase-phosphatase
**PRD**	PIKK regulatory domain
**PRKAA2**	Protein kinase, AMP-activated, α 2 catalytic subunit
**PRRs**	Pattern recognition receptors
**PTBP1**	Polypyrimidine tract-binding protein 1
**PTEN**	Chromosome ten
**PTPRJ**	Protein tyrosine phosphatase receptor type J
**Pum2**	Pumilio RNA binding family member 2
**PYGO2**	Pygopus 2
**RAD18**	Radiation Sensitive 18
**RAD51**	Radiation Sensitive 51
**RAS**	Rat sarcoma
**RCC**	Renal cell carcinoma
**RNAi**	RNA interference
**ROCKs**	Rho-associated coiled-coil-containing protein kinases
**ROS**	Reactive oxygen species
**RPN6**	The ribophorin family 6
**RRM2**	Ribonucleotide reductase subunit M2
**rRNA**	Ribosomal RNA
**RRP9**	Ribosomal RNA Processing 9
**RS**	Replication stress
**SETDB1**	SET domain bifurcated 1
**SiMGMT**	Small interfering O6 methylguanine DNA methyltransferase RNA
**siRNAs**	Small interfering RNA
**SMAD1**	SMAD family member 1
**SMARCA5**	An ATPase of the ISWI class of chromatin remodeler
**snoRNAs**	Small nucleolar RNA
**Sp1**	Specific protein 1
**SSBR**	Single strand break repair
**ssDNA**	Single stranded DNA
**SSRP1**	Structure-specific recognition protein 1
**TCR**	Transcriptional coupled repair
**TDP1**	Tyrosine DNA phosphodiesterase 1
**TMZ**	Temozolamide
**TNBC**	Triple negative breast cancer
**TOP1**	Topoisomerase I
**TOP2A**	Type II topoisomerases
**TRAF6**	Tumor necrosis factor receptor-associated factor 6
**TRB3**	Tribbles homologue 3
**TSCC**	Tongue squamous cell carcinoma
**TTD**	Lignosulphia malnutrition
**VN1R5**	Vomeronal type 1 receptor 5
**XP**	Xeroderma pigmentosum
**XPC**	Xeroderma pigmentosum group C
**ZCCHC4**	Znc finger CCHC domain containing protein 4
**131I**	Iodine-131
**3′- UTR**	3′- untranslated region
**5-FU**	5-fluorouracil
**γH2AX**	Phosphorylation of histone H2AX
