# Supplementary material for: DNA damage response-related ncRNAs as regulators of therapy resistance in cancer
Source: Front Pharmacol. 2024 Aug 26;15:1390300. doi: 10.3389/fphar.2024.1390300 (PMC11381396; doi:10.3389/fphar.2024.1390300)
Supplement: Supplementary file 1 [file Table1.docx]

| **Supplementary Table 1. Regulation of ncRNAs in cancer chemotherapy resistance** | | | | | | | |
| --- | --- | --- | --- | --- | --- | --- | --- |
| **ncRNAs** | **Expression in tumor** | | **Role in cancer** | **Related drugs** | **Related genes or pathways** | **Tumor types** | **Refs** |
| **MiRNAs** | | | | | | | |
| miR-363 | | Downregulated | tumor suppressor | Cisplatin | Mcl-1 | Hepatocellular Carcinoma | 59 |
| miR-769-5p | | Upregulated | oncogene | Cisplatin | CASP9, NEDD4L | Gastric cancer | 60 |
| miR-203 | | Upregulated | tumor suppressor | Cisplatin | DJ-1, PTEN, PI3K/AKT pathway | Pancreatic cancer | 61 |
| miR-144 | | Downregulated | tumor suppressor | 5-Fluorouracil | Nrf2 | Hepatocellular Carcinoma | 62 |
| miR-567 | | Downregulated | tumor suppressor | 5-Fluorouracil | PIK3AP1, PI3K/AKT pathway, c-MYC | Gastric cancer | 63 |
| miR-27a-5p | | Upregulated | tumor suppressor | Doxorubicin | RAS, MEK, FOS, PTEN, AKT, SMAD1 | Gastric cancer | 64 |
| **LncRNAs** | | | | | | | |
| LINC-PINT | | Downregulated | tumor suppressor | Cisplatin | EZH2, ATG5 | Gastric cancer | 74 |
| LINC00680 | | Upregulated | oncogene | 5-Fluorouracil | miR-568, AKT3 | Hepatocellular Carcinoma | 75 |
| OVAAL | | Upregulated | oncogene | 5-Fluorouracil | pyrimidine biosynthesis | Gastric cancer | 76 |
| PDIA3P1 | | Upregulated | oncogene | Doxorubicin | hMTR4, miR125/124, TRAF6 | Hepatocellular Carcinoma | 77 |
| **CircRNAs** | | | | | | | |
| circMRPS35 | | Upregulated | oncogene | Cisplatin | circMRPS35-168aa | Hepatocellular Carcinoma | 87 |
| circUBAP2 | | Upregulated | oncogene | Cisplatin | miR-300, ASF1B | Breast cancer | 88 |
| circCPM | | Upregulated | oncogene | 5-Fluorouracil | PRKAA2 | Gastric cancer | 89 |
| circ_0003998 | | Upregulated | oncogene | Doxorubicin | miR-218-5p, EIF5A2 | Hepatocellular Carcinoma | 90 |
| circCUL2 | | Downregulated | tumor suppressor | Doxorubicin | miR-142-3p, ROCK2 | Gastric cancer | 91 |
| **Others** | | | | | | | |
| piR-17560 | | Upregulated | oncogene | Docetaxel | FTO-mediated m6A demethylation | Breast cancer | 98 |
| piR-39980 | | Downregulated | oncogene | Doxorubicin | drug accumulation | Fibrosarcoma | 99 |
| SNORD1C | | Upregulated | oncogene | 5-Fluorouracil | Wnt pathway | Colorectal cancer | 101 |
| SNORD3A | | Upregulated | oncogene | 5-Fluorouracil | miR-185-5p | Breast cancer | 102 |
| si-Rac-1 | | Upregulated | Tumor suppressor | Cisplatin | Rac-1 | Breast cancer | 105 |
