# Supplementary material for: DNA damage response-related ncRNAs as regulators of therapy resistance in cancer
Source: Front Pharmacol. 2024 Aug 26;15:1390300. doi: 10.3389/fphar.2024.1390300 (PMC11381396; doi:10.3389/fphar.2024.1390300)
Supplement: Supplementary file 2 [file Table2.docx]

| **Supplementary Table 2. Regulation of ncRNAs in cancer radiotherapy resistance** | | | | | |
| --- | --- | --- | --- | --- | --- |
| **ncRNAs** | **Expression in tumor** | **Role in cancer** | **Related genes or pathways** | **Tumor types** | **Refs** |
| **MiRNAs** | | | | | |
| miR-96-5p | Downregulated | Oncogene | PTEN | Head and neck squamous cell carcinoma | 65 |
| miR-4443 | Upregulated | Tumor suppressor | PTPRJ | Esophageal squamous cell carcinoma | 66 |
| miR-621 | Downregulated | Tumor suppressor | SETDB1 | Hepatocellular caricinoma | 67 |
| miR-410 | Upregulated | Oncogene | PTEN, PI3K, mTOR | Non-small cell lung cancer | 68 |
| miR-450a-5p | Upregulated | Tumor suppressor | DUSP10 | Esophageal squamous cell carcinoma | 69 |
| **LncRNAs** | | | | | |
| HOTAIRM1 | Upregulated | Oncogene | Mitochondrial function; ROS | Glioblastoma cells | 79 |
| DNM3OS | Upregulated | Oncogene | DNA damage | Endometrial cancer | 80 |
| lncTUG1 | Upregulated | Oncogene | MET, EGFR, AKT | Esophageal squamous cell carcinoma | 81 |
| HNF1A-AS1 | Upregulated | Oncogene | miR-92a-3p | Non-small cell lung cancer | 82 |
| LINC00518 | Upregulated | Oncogene | miR-33a-3p, HIF-1α | Melanoma | 83 |
| **CircRNAs** | | | | | |
| circRNA_100367 | Downregulated | Oncogene | miR-217, Wnt3 pathway | Esophageal squamous cell carcinoma | 92 |
| circux1 | Upregulated | Oncogene | Caspase1 | Hypopharyngeal squamous cell carcinoma | 93 |
| circ-ACAP2 | Upregulated | Oncogene | miR-143-3p, FZD4 | Colorectal cancer | 94 |
| circMTDH.4 | Upregulated | Oncogene | miR-630, AEG-1 | Non-small cell lung cancer | 95 |
| circVRK1 | Downregulated | Tumor suppressor | miR-624-3p, PTEN, PI3K/AKT pathway | Esophageal squamous cell carcinoma | 96 |
| **Others** | | | | | |
| si-GLUT-1 | Upregulated | Tumor suppressor | GLUT-1 | Laryngeal cancer | 104 |
